# An Artificial Intelligence Analysis of Electrocardiograms for the Clinical Diagnosis of Cardiovascular Diseases: A Narrative Review

**DOI:** 10.3390/jcm13041033

**Published:** 2024-02-11

**Authors:** Assunta Di Costanzo, Carmen Anna Maria Spaccarotella, Giovanni Esposito, Ciro Indolfi

**Affiliations:** 1 Division of Cardiology, Cardiovascular Research Center, University Magna Graecia Catanzaro, 88100 Catanzaro, Italy; 2 Division of Cardiology, Department of Advanced Biomedical Sciences, University of Naples Federico II, 80126 Naples, Italy; carmenannamaria.spaccarotella@unina.it (C.A.M.S.);

**Keywords:** artificial intelligence, deep learning, convolutional neural networks, electrocardiogram, cardiovascular diseases

## Abstract

Artificial intelligence (AI) applied to cardiovascular disease (CVD) is enjoying great success in the field of scientific research. Electrocardiograms (ECGs) are the cornerstone form of examination in cardiology and are the most widely used diagnostic tool because they are widely available, inexpensive, and fast. Applications of AI to ECGs, especially deep learning (DL) methods using convolutional neural networks (CNNs), have been developed in many fields of cardiology in recent years. Deep learning methods provide valuable support for rapid ECG interpretation, demonstrating a diagnostic capability overlapping with specialists in the diagnosis of CVD by a classical analysis of macroscopic changes in the ECG trace. Through photoplethysmography, wearable devices can obtain single-derivative ECGs for the recognition of AI-diagnosed arrhythmias. In addition, CNNs have been developed that recognize no macroscopic electrocardiographic changes and can predict, from a 12-lead ECG, atrial fibrillation, even from sinus rhythm; left and right ventricular function; hypertrophic cardiomyopathy; acute coronary syndromes; or aortic stenosis. The fields of application are many, but numerous are the limitations, mainly associated with the reliability of the acquired data, an inability to verify black box processes, and medico-legal and ethical problems. The challenge of modern medicine is to recognize the limitations of AI and overcome them.

## 1. Introduction

Cardiovascular disease is the leading cause of death in industrialized countries, accounting for 32% of all deaths and 38% of deaths under the age of 70 (data from the World Health Organization on cardiovascular diseases). Although the proper management of heart disease reduces the incidence of mortality, great emphasis is placed on the early recognition, prevention, and, when necessary, timely treatment of cardiovascular disease. 

Electrocardiograms (ECGs) are the cornerstone form of examination in cardiology and are the most widely used diagnostic tool for assessing cardiac diseases, with the advantage of being widely available, inexpensive, painless, and easily performed. An electrocardiogram is a graphic reproduction of the electrical activity of the heart recorded on the surface of the body. The term electrocardiogram was introduced by Einthoven in 1893, who was awarded the Nobel Prize in Medicine for this discovery. This tool records the activity of millions of individual cardiomyocytes graphically, quickly, and easily. The interpretation of an ECG requires a high degree of expertise and evaluation by a cardiologist. Classical evaluation by the specialist makes it possible to diagnose specific clinical disorders through a macroscopic assessment of changes in individual segments, such as atrial fibrillation (AF), ventricular hypertrophy, or acute coronary syndromes (ACS) with ST-segment changes. 

The introduction to medicine of artificial intelligence (AI), and especially of deep learning (DL) and convolutional neural networks (CNNs or ConvNets), has proven to be a great support for healthcare professionals in improving the quality of care. ECGs are an ideal substrate for deep learning AI applications since they are widely available and yield reproducible raw data that are easy to store and transfer in a digital format. The application of AI to ECGs is an opportunity to support human intelligence, reduce errors, improve interpretation, and obtain more information [1]. It makes possible the identification and diagnosis of situations that cannot be recognized by traditional ECG reading. The application of AI to cardiology is a useful tool to automate and speed up diagnosis in urgent situations, for example, myocardial infarction (MI) [2]. In addition, it can be used as a valuable screening tool for AF. Of great interest is the application of AI to wearable devices [3,4]. Smart watches with AI could be used in the future for the assessment of oxygen saturation, QT intervals, or Brugada syndrome [5,6,7]. An interesting area of interest will be the use of smart watches with AI for the reliable diagnosis of myocardial infarction using a multichannel electrocardiogram approach [8]. Above all, AI has become capable of extracting additional information that cannot be assessed with classical diagnostic criteria, such as in the case of the diagnosis of severe aortic stenosis using TP and U-wave analysis [9].

This innovation still has limitations, mainly associated with the reliability of the acquired data, non-validatable embedded biases, the impossibility of verifying the machine’s decision-making processes, and ethical problems. The challenge for modern medicine is to recognize these limitations and overcome them.

## 2. Artificial Intelligence, Machine Learning, and Deep Learning

Artificial intelligence is radically changing the modern world, and its great potential in the field of medicine is certainly a challenge for science and health. In particular, the last decade has seen a growing interest in the application of artificial intelligence to cardiovascular medicine. 

**Figure 1:**
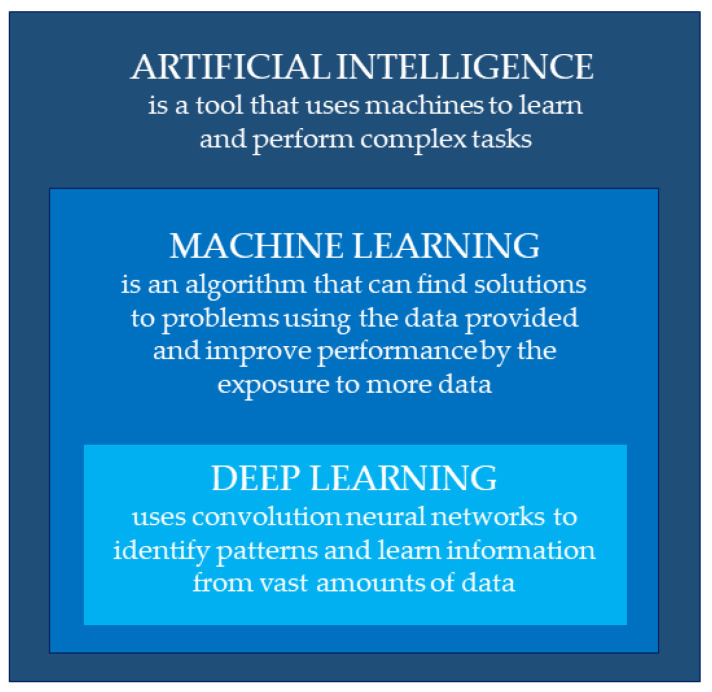
Definitions of artificial intelligence, machine learning, and deep learning.

Artificial intelligence is a tool that uses machines to learn and perform complex tasks and improve performance through experience (Figure 1).

ML is an algorithm that can find solutions to problems (patterns) using the data provided. Whereas traditional learning learns from rules, ML learns from examples. The machine is given a task to perform and examples in the form of input (features) and output (labels). The computer, in this way, finds the pattern that links the input and output through a process called machine learning. Machine learning can take place in two ways: supervised or unsupervised. Supervised learning is based on a defined input and output, whereas unsupervised learning only receives input. These features of machine learning have a very high potential, as they allow for the analysis of a huge amount of data and for finding statistical patterns—a performance that cannot be fulfilled by human intelligence alone. 

However, machine learning has a limited capacity to learn, being strictly dependent on features provided by experts. This limitation is overcome by deep learning, a sub-category of machine learning, which improves its performance by accessing large amounts of data and allows it to extract information that cannot be evaluated from a human perspective. DL uses neural networks (NNs) to identify patterns in data or to make predictions. It analyses many simple linear patterns (nodes) arranged in series (layers) to create a hierarchical structure: each layer, composed of simple entities, serves to represent complex information by passing through one or more hidden layers. In this way, deep learning learns extremely complex relationships between features and labels.

Convolutional neural networks are used in the analysis and interpretation of images (e.g., electrocardiograms). Such networks exploit principles of linear algebra, in particular matrix multiplication, to identify patterns within an image. In general, it consists of an input block; one or more hidden blocks (hidden layers), which perform calculations using activation functions; and an output block, which performs the actual classification. The difference is the presence of the three additional layers: a convolutional layer, a pooling layer, and a fully connected (FC) layer. The convolutional level requires input data (expressed as a matrix), a feature detector (filter or kernel), and a feature map. This layer extrapolates the characteristics (features) of the image via the filter. The greater the number of filters, the greater the complexity of the features that can be detected. The pooling layer applies a filter to the entire output. It performs an aggregation of the information, generating feature maps of a smaller size. Typically, each convolutional layer is followed by a pooling layer to reduce the size of the matrix to recognize increasingly more precise image features. Finally, the FC layer performs the classification task based on the features extracted from the previous layers and their different filters. In this way, the inputs are classified appropriately [10].

## 3. Application Fields in Electrocardiography

The electrocardiogram is the cornerstone form of examination in cardiology. The ECG consists of several waves that, in normal conditions, repeat in the same order in each cardiac cycle. Einthoven assigned the letters of the alphabet “P, Q, R, S, T” to the various waves, starting arbitrarily from the letter P, which had already been used by Descartes to indicate the points of a curve in his studies on refraction. The P wave corresponds to atrial depolarization, the QRS complex corresponds to ventricular depolarization, and the T wave represents ventricular repolarization, followed by the U wave, whose genesis is still debated. The classical electrocardiogram comprises 12 leads, which describe the depolarization and repolarization processes from different observation points: six leads are recorded with electrodes placed on the limbs (unipolar and bipolar peripheral leads) and six are obtained with electrodes placed in the precordial region (precordial leads). By recognizing abnormalities compared to the normal ECG, it is possible to diagnose numerous cardiovascular diseases: conduction defects, arrhythmias, myocardial infarction, dysionia, cardiomyopathies, and pericarditis. Correctly and accurately interpreting an ECG is therefore an indispensable prerogative of cardiology. However, around 300 million ECGs are recorded every year, making it difficult for cardiologists to meet the demand for reporting [11]. 

Furthermore, misdiagnosis, especially by less experienced professionals, can lead to inappropriate clinical decisions or adverse events for patients. Bogun et al. described the adverse clinical consequences associated with the misdiagnosis of atrial fibrillation. Starting anticoagulant treatment when unnecessary is potentially harmful, as it exposes the patient to an increased risk of bleeding [12]. Therefore, the accurate reading of the ECG is a great interest topic, and in recent decades, automatic diagnosis has been studied worldwide with large databases. The electrocardiogram is an ideal examination for AI, as it is an instrument that contains countless raw data that can be easily transformed into mathematical and digital languages. The application of artificial intelligence is an opportunity to support human intelligence: to help the healthcare provider by pointing out aspects of the images that deviate from the norm, to automate and speed up diagnosis, and to extract additional information that cannot be evaluated using classic diagnostic criteria [13]. 

Initially, machine learning was applied to the automatic diagnosis of ECG abnormalities, but achieved results limited by data quality, with a classification performance of 71.6–74.2% [14]. 

Substantially greater results have been achieved with deep learning methods and CNNs. Initially, several studies demonstrated the application of deep learning for the diagnosis of AF in single-lead ECGs [15,16]. This capability paved the way for the development of wearable devices capable of diagnosing AF or cardiac arrhythmia from a single-lead ECG acquired by photoplethysmography. 

The application of CNNs to the analysis of 12-lead ECGs opened new opportunities for scientific progress in the field of electrocardiography [17]. Deep learning has demonstrated a superior diagnostic capability compared to cardiologists for the detection of different categories of arrhythmias. In support, Hannun et al. described the superiority of CNNs over cardiologists in diagnosing 12 classes of arrhythmias in 12-lead ECGs [18]. 

A previous study applied a CNN model for the correct interpretation and diagnosis of 21 rhythm classes on 828 ECGs (12-lead of 10 s duration) [19]. The deep learning model was trained on 135,817 training ECGs (training dataset) and 17,955 validation ECGs (validation dataset). The interpretation of the ECGs was also performed by clinical cardiologists divided into three categories of clinical experience. The network consisted of an input represented by a 5000 × 12 matrix, 15 convolutional layers, and an output formed of a 1 × 21 vector: each element of the vector represented a type of ECG rhythm. Zhu et al. described that the deep learning model achieved a higher number of accurate diagnoses (80%) than the average performance of cardiologists (67%, 69%, and 75%, according to their clinical experience category).

Furthermore, the combination of data obtained from the CNN with expert features (statistical features, signal procession features, and medical features) further improved the performance of neural networks [20].

Since 2019, deep learning has made it possible to expand the number of diagnoses possible from the analysis of heart rhythms using the classical electrocardiogram. By eliminating evaluations based on coarse patterns, deep learning only analyses data and draws conclusions that exceed human capacity alone. Specifically, CNNs interpret ECGs quicker than human evaluation. They also detect conditions that are unrecognizable by human interpreters. In this way, the ECG becomes a powerful non-invasive biomarker.

Its current fields of application are associated with the diagnosis of arrhythmias (e.g., atrial fibrillation, ventricular tachyarrhythmias), valvulopathies (aortic stenosis and mitral insufficiency), heart failure, extracardiac evaluation (hyperkalemia, anemia, health status), cardiomyopathies (as hypertrophic cardiomyopathies), myocardial infarction, pulmonary hypertension, and channelopathies. In addition, numerous studies are investigating the ability of AI to predict cardiovascular diseases in order to improve prevention and early diagnosis and prevent the irreversible progression of cardiovascular diseases. Fields of study are paroxysmal atrial fibrillation, aortic stenosis, mitral insufficiency, heart failure with a preserved ejection fraction, and critical patient states (e.g., cardiac arrest and deterioration) (Table 1).

## 4. Atrial Fibrillation

Atrial fibrillation is associated with an increased risk of stroke, heart failure, and mortality. According to the current ESC 2020 guidelines, the diagnosis is made by finding AF on a 12-lead ECG, cardiac telemetry, Holter ECG, or implanted loop devices [44]. However, the diagnosis can be elusive because about 33% of patients are asymptomatic and 25% have atypical symptoms [45]. Furthermore, atrial fibrillation can be intermittent or paroxysmal; about 15% of patients with a “cryptogenic stroke” have paroxysmal AF [46]. Artificial intelligence-enabled electrocardiography could be a great resource to identify AF undetected by testing, with great clinical, therapeutic, and prognostic impacts. Some studies have evaluated the P-wave characteristics of a sinus rhythm ECG to predict the diagnosis of AF. These features have been defined as the electrocardiographic signature of AF during sinus rhythm. Attia et al. trained a deep learning model to find such electrocardiographic signs, which might not be seen by the human eye. The model proved able to identify the presence of AF (AUC: 0.87) [21]. An article published in *Circulation* analyzed more than 1.5 million 12-lead ECG traces and predicted the new onset of AF at 1 year with a sensitivity of 69% and a specificity of 81% [22]. The limitations of this field of interest relate to the therapeutic approach. Currently, the predictive ability of AI-ECG is 21.5% at 2 years and 52.2% at 10 years [47]. Higher thresholds in the future might justify the initiation of anticoagulant therapy in patients at high risk of AF onset. The BEAGLE study (NCT04208971), the first randomized, controlled clinical trial, is underway to validate an artificial intelligence algorithm capable of identifying patients with a high probability of AF from ECG analysis [48].

In patients with suspected AF, one strategy may be the implantation of an implantable loop recorder (ILR). Of these, the most studied is the Reveal LINQ system (Medtronic), which has an AF detection algorithm based on R-wave irregularities and P-wave discrimination. This algorithm was evaluated in the Reveal XT Performance Trial, which demonstrated a sensitivity of 96.1% and a specificity of 85.4% [49]. Such devices, however, are implanted invasively. Hygrell et al. demonstrated that an AI algorithm can predict AF even on single-lead ECGs in sinus rhythm, especially in older subjects [50]. The development of a deep learning algorithm for recognizing AF from a single-lead ECG could simplify screening, as single-lead ECGs are easily obtained via wearable devices. Photoplethysmography is more contemporary and has been used in some wearable devices including watches, wrist straps, and smartphones. The mechanism is based on a light directed at the skin and a photo sensor [23,24,51,52]. This technique, combined with a deep neural network, has been evaluated in several clinical studies to detect AF from smart watch data. Tison et al. conducted a small demonstration study to evaluate the ability of passive AF detection by photoplethysmography technology combined with a deep neural network. The smart watch algorithm achieved a sensitivity of 98% and a specificity of 90.2% in detecting AF in a cohort of 51 patients [25]. Bumgarner et al. tested an algorithm for automatic AF detection using an Apple Watch accessory. The Kardia Band (KB) supplied by AliveCor is a band that can record a single-lead ECG and transmit it to an Apple smart watch via Bluetooth. In the study, the results obtained by the KB detection algorithm were compared to physician-interpreted 12-lead ECGs. A total of 100 patients were enrolled to undergo cardioversion. The KB interpretations showed excellent agreement with the reported ECGs and demonstrated a sensitivity of 93% and a specificity of 84% in diagnosing AF [3]. In turn, Wasserlauf et al. compared the accuracy of AF detection using the Apple Watch algorithm with the Kardia Band in patients with ILR. The CNN detected AF with a sensitivity of 97.5%, detecting 80 episodes of AF compared to 82 detected by ILR [4].

## 5. Aortic Stenosis

Aortic valve stenosis (AVS) is the most frequent heart valve disease in the general population (0.4%) and affects 2% of individuals after the age of 65 years and 12% after the age of 75 years [53]. The prognosis is poor, and the onset of symptoms is associated with a survival rate of 50% at 2-year follow-up [54]. At present, the only effective therapy for severe aortic stenosis is valve replacement by surgery or transcatheter aortic valve implantation (TAVI), which has made it possible to treat patients who are elderly or deemed inoperable [55,56]. Currently, the gold standard for the diagnosis and quantification of AVS is echocardiography [57]. The development of a reliable screening tool to detect significant AVS is important because most patients with severe AVS are asymptomatic and early diagnosis is essential to prevent irreversible disease progression and mortality. If significant AVS could be detected using a conventional 12-lead ECG or a single-lead device, patients could be referred for echocardiography and early diagnosis. This need also arises from a growing body of data supporting early treatment even in asymptomatic patients [58]. However, no reliable screening tools currently exist. Classical 12-lead ECG analysis does not allow for the recognition of aortic stenosis. Electrocardiographic evidence of left ventricular hypertrophy, using the Sokolow index, is not diagnostic and does not allow for a differential diagnosis with other pathologies (e.g., aortic insufficiency) [59]. Recently, the novelty of artificial intelligence has made it possible to correlate electrocardiographic changes in a cluster of patients with manifest aortic stenosis or a predisposition for valve disease. Two studies conducted on geographically and ethnically various populations demonstrated the great potential of AI applied to ECGs for screening and diagnosing valve disease. Deep learning algorithms showed that the TP interval and U waves in the right precordial leads were the most weighted for determining the presence of AVS. Kwon et al. developed a deep learning algorithm to detect aortic stenosis using 12-lead and single-lead ECGs. The algorithm for screening aortic stenosis achieved an AUC of 0.86–0.88 and a negative predictive value >99% [26]. The same authors tested an algorithm for the detection of mitral insufficiency, with promising results [28]. 

**Figure 2:**
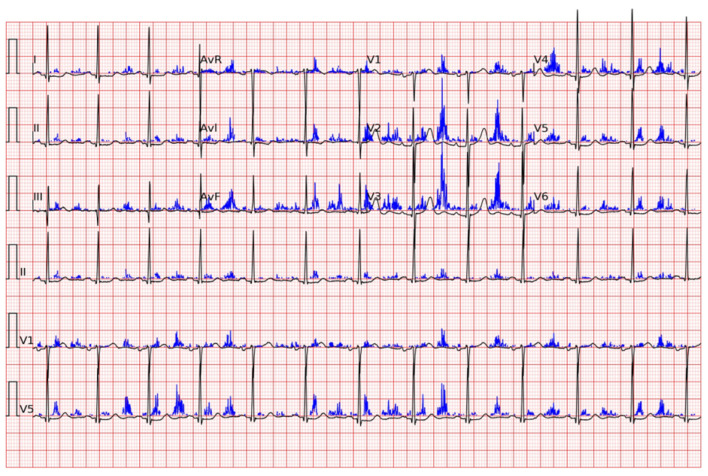
A representative electrocardiogram example for true positive is shown. The probability of moderate or severe aortic stenosis by artificial intelligence electrocardiogram is 0.92 in the presented case. The blue lines are the “saliency” guiding the selection of attended locations.

Cohen Shelly et al. developed a deep learning algorithm capable of recognizing moderate-to-severe aortic stenosis (AUC: 0.85) in asymptomatic subjects with a high sensitivity and specificity, but, above all, a high negative predictive value (VPN 99%). They also demonstrated the algorithm’s superiority in recognizing asymptomatic subjects, compared to physicians able to make a diagnosis based on an auscultation of the murmur (only 39% of physicians were able to recognize the murmur) [9] (Figure 2). 

This material was originally published in “Electrocardiogram screening for aortic valve stenosis using artificial intelligence” by Cohen-Shelly Michal et al., edited by the *European Heart Journal*, and has been reproduced with permission from Oxford University Press.

A more recent, retrospective study by Harmon et al. also demonstrated the ability of the AI-ECG algorithm to predict disease progression by analyzing the TP interval and T/U-wave morphology [27].

## 6. Ventricular Dysfunction

Left ventricular dysfunction includes different phenotypes: decompensation with a reduced ejection fraction (LVEF < 40%), decompensation with a moderately reduced ejection fraction (LVEF 41–49%), and decompensation with a preserved ejection fraction (associated with the finding of symptoms and signs of decompensation, diastolic dysfunction, and LVEF > 50%. An estimated 6% of the general population has an undiagnosed, asymptomatic left ventricular ejection fraction (LVEF < 50%)) [60]. Transthoracic echocardiography (TTE) is the gold standard for assessing left ventricular function, but it is an expensive test and not always easy to perform. The atrial natriuretic peptide assay is a simple screening test but is invasive, as it requires a blood sample [61]. 

The 12-lead resting electrocardiogram is known to have low sensitivity and a low positive predictive value for left ventricular systolic dysfunction. There is currently no low-cost, non-invasive test for screening left ventricular dysfunction. In 2010, Schlegel and colleagues evaluated an advanced 12-lead ECG test capable of evaluating seven electrocardiographic parameters combined into computerized multivariate scores. This improved the specificity, sensitivity, and positive predictive value of the ECG in recognizing left ventricular dysfunction [62].

The application of artificial intelligence to ECGs for recognizing changes associated with ventricular dysfunction could be useful for tracking patients at risk.

Attia et al. trained a neural network with a total of 97,829 ECG–TTE pairs. The network was shown to correctly recognize patients with left ventricular dysfunction out of an independent group of 52,870 ECGs. The statistical analysis obtained an AUC of 0.93, a sensitivity of 93.0%, a specificity of 86.3%, and an accuracy of 85.7%. Furthermore, among “false positive” patients (i.e., with EF judged abnormal by the network, but normal at TTE), the risk of developing ventricular dysfunction was increased by 10% at 5 years [29]. Its use was also tested for patients presenting to the emergency department with acute dyspnea. Dyspnea can have multifactorial causes, and the correct identification of the cause guides the physician towards the right treatment. In the 1606 patients included, the algorithm correctly diagnosed left ventricular dysfunction with an accuracy of 85.9% for LVEF < 35% and 86% for LVEF < 50% [30]. Vaid et al. applied a deep learning algorithm to identify left and right ventricular dysfunction, with encouraging results [31]. A randomized, controlled clinical trial, the EAGLE study (NCT04000087), is underway to screen for left ventricular dysfunction by analyzing 12-lead ECGs with deep learning systems [63]. A recent study also demonstrated the possibility of screening for HFrEF from ECGs, with very encouraging accuracy data [64]. Unfortunately, at external validation, the accuracy of the data was lower, with more false positives; specifically, in the ECG subgroups with tachycardia, atrial fibrillation, and conduction delays, the AUC curves were lower [65].

## 7. Cardiomyopathies

Hypertrophic cardiomyopathy (HCM) is associated with an annual incidence of cardiovascular death (sudden cardiac death, heart failure, and thrombo-embolism) of 1–2% [66]. This value is 10% in the pediatric population, with a risk of sudden cardiac death (SCD) of 1.2–1.5% [67]. According to the recent ESC 2023 guidelines on the diagnosis of hypertrophic cardiomyopathy, the diagnostic criteria are mainly echocardiographic with a finding of LV wall thickness ≥15 mm [68]. The screening method to calculate the risk of SCD at 5 years (Class IB) is the “HCM Risk-SCD calculator”. However, early diagnosis through the detection of electrocardiographic alterations could be useful for asymptomatic patients and could simplify diagnosis with a non-invasive, quick, and simple tool such as an ECG. More than 90% of patients have ECG changes, but these do not allow for a differential diagnosis by the clinician as they are non-specific. A 2015 paper first demonstrated the diagnosis of HCM by 12-lead ECG by classifying heartbeats using machine learning methods [32].

A recent study evaluated a deep learning algorithm for HCM diagnosis from a 12-lead ECG. It demonstrated a negative predictive value of 99%, a sensitivity of 87%, a specificity of 91%, and an AUC of 0.96 [33]. This model applied to the general population could improve screening for HCM. Tison et al. developed a deep learning model for the detection of four diseases, such as HCM, pulmonary hypertension (PAH), cardiac amyloid (CA), and mitral valve prolapse (MVP), using ECG profiles at 12 detection points. The model was able to discriminate PAH (AUC: 0.94) and HCM (AUC: 0.91) promisingly, while weaker discrimination was found for CA (AUC: 0.86) and MVP (AUC: 0.77) [34].

## 8. Myocardial Infarction and Ischemic Cardiomyopathy

Acute coronary syndromes (ACSs) are associated with high mortality and morbidity, being the leading cause of death worldwide. Therefore, early diagnosis and medical intervention with appropriate treatment are crucial to reduce the risks for the patient. The latest ESC guidelines on the management of ACS indicate 12-lead ECG as the first-line diagnostic tool in patients with chest pain (or equivalent signs/symptoms of angina) to be performed within 10 min. The main electrocardiographic changes of myocardial infarction are ST-segment elevation, T-wave inversion, or the appearance of necrotic Q waves. However, the diagnosis of STEMI is defined by the ECG finding of ST-segment elevation at the J point in at least two contiguous leads [69]. Several studies have been conducted using single-lead (II-lead) ECG datasets applying a CNN. Among them, Acharya et al. used an 11-layer CNN to automatically diagnose MI. The results obtained on single-lead ECGs were an accuracy of 95.22%, a sensitivity of 95.49%, and a specificity of 94.19% [35]. Studies conducted on 12-lead ECGs enabled the automatic detection and localization of MI by a CNN [36,37,38,70,71]. In one of these, Chen et al. trained a CNN to recognize and localize myocardial infarction from 12-lead ECGs. A total of 15,285 ECGs were used as the training set and 6552 ECGs as the validation set. Finally, 205 ECGs were used as the testing set and demonstrated an accuracy of 82.7% [2]. Furthermore, Tadesse et al. were able to implement the diagnosis with information regarding the time of onset of MI, classifying the event as acute, recent, or long-standing [72].

Chen et al. proposed an automatic ST-segment elevation detection system using ECGs performed in an ambulance to speed up diagnosis and support decision making at triage. The goal was to reduce the time to diagnosis, so the response time, defined as the time interval between ECG transmission and interpretation, was analyzed. The system was analyzed and interpreted, with excellent results of an AUC, accuracy, precision, and specificity of 0.997, 0.992, 0.889, and 0.994. It also demonstrated a reduction in diagnostic delays, with a response time inferior to that of physicians (37.2 ± 11.3 vs. 113.2 ± 369.4 s, *p* < 0.001) [39].

Chronic coronary syndromes must also be diagnosed early. The early initiation of appropriate treatment helps to prevent the development of myocardial scarring and, subsequently, ischemic heart disease [73]. The gold standard for the diagnosis of myocardial scarring (MS) is nuclear magnetic resonance imaging (MRI) by identifying scar tissue using gadolinium. However, the costs of MRI and the small number of specialists for reporting make the use of this tool in the diagnosis of MS limited. Gumpfer et al. proposed a deep learning model to detect MS from 12-lead ECGs. ECG and MRI data were collected on 114 patients. The CNN model recognized MS with an accuracy of 78.0%, a sensitivity of 70.0%, and a specificity of 84.3% [40].

## 9. Electrolyte Abnormalities

One of the main electrolytes involved in the process of cardiac depolarization and repolarization is potassium (K+). Pathological variations in potassium can be recognized by analyzing the ECG tracing. Hyperkalemia occurs mainly in cases of renal failure, acidosis, or poor therapy management. Electrolyte alterations are subtle and difficult to recognize because they are frequently asymptomatic. Serum potassium dosage allows hyperkalemia to be classified as mild (5–5.5 mEq/L), moderate (5.5–6 mEq/L), or severe (>6 mEq/L) [41]. Such an increase in extracellular K concentrations (usually, serum changes >6 mEq/L) is associated with trace alterations such as an increased T-wave voltage (peaking of T waves), P-wave and PR changes (such as PR shortening), and QRS prolongation. The risk associated with hyperkalemia is the initiation of ventricular fibrillation and the death of the patient. According to the ESC 2021 guideline on heart failure in patients at risk of developing dysionia, especially in patients with renal failure, frequent monitoring of blood tests should be performed to control and stabilize K+ and creatinine [74,75]. This indication is not easy to implement, and blood tests are a simple but minimally invasive tool for the patient. The application of deep learning systems to the ECG can be used to screen hyperkalemia and reduce the risk of fatal arrhythmias. Galloway et al. trained a CNN to recognize pathological potassium levels from the analysis of the ECG trace. They defined hyperkalemia as a K+ value ≥ 5.5 mEq/L and analyzed more than 1.5 million 12-lead ECGs using a CNN with 11 convolutional layers. The performance of the CNN proved to be good for the diagnosis of hyperkalemia, with a negative predictive value of 99%, an AUC of 0.853–0.883, and a sensitivity of 88.9–91.3% [41]. Lin et al. applied an 82-layer convolutional deep learning algorithm to detect alterations in serum potassium. Hypokalemia is associated with lower-than-normal serum potassium levels and ECG alterations such as PR prolongation, ST-segment depression, a T-wave decrease in voltage until disappearance or T-wave inversion, QTc prolongation, and U-wave appearance. The deep learning model performed better than physicians in detecting dysionia and had a sensitivity result of 84.5–95.6% [42]. Attia et al. described the possibility of potassium measurements without blood samples, only with the use of single-lead ECGs. This study, based on T-wave morphology, excluded patients with biphasic, bimodal, or inverted T waves. Estimates of blood potassium levels were obtained with an average error of 0.5 ± 0.42 mmol/L. This could lead to the development of wireless, remote, continuous, and non-invasive monitoring technologies with the ability to monitor the trend and send alerts to patients at risk of fatal arrhythmias, such as renal failure or dialysis [43].

## 10. Obstacles and Challenges to Overcome in Artificial Intelligence

The potential of AI applied to medicine is numerous, which is why close monitoring of the system’s results and methodology is necessary. Ethical limitations, raw input data, incorrect input, overfitting, and a lack of interpretability of the decision-making process could be obstacles in the routine use of AI systems [76]. 

As described by Attia et al., the main problems are related to explainability, uncertainty, and robustness (Table 2) [13].

Research is focusing on making the mechanism of black boxes explainable so that the pattern by which inputs generate outputs is known. The inability to monitor and correct the risk of unreasonable decisions leads to an important ethical problem: who is responsible for a diagnosis that the medical professional has no way of verifying? One example is the automatic diagnosis of new continuous heart rhythm monitoring devices. The verification of a diagnosis for devices that record for 24–48 h is rapid. The verification of a continuous recording, weekly or monthly, is impossible. The diagnosis of the absence or presence of AF or other rhythm disturbances implies therapeutic choices based on uncontrolled decision making. Explainable artificial intelligence would make the machine’s decision-making process known, allowing ethical problems to be overcome. The quantification of uncertainty is crucial to increase confidence in the results obtained. 

The uncertainty error is related to the training input data. In particular, the use of raw data increases the amount of noise. Normally, deep learning provides outputs by analyzing the input data provided. Chen et al. described safe learning through uncertainty estimation, which enables a better evaluation of noisy data. Through trained AF detection models, deep learning has demonstrated a superior classification capability for raw data (ECG performed in intensive care) [77]. Another problem related to supervision is datasets with large numbers of ECGs. Including hundreds of thousands, or more, of ECGs, as in most existing publications, makes it difficult to perform quality control on each individual ECG. Therefore, the system may present results affected by reduced signal quality. Therefore, noise, missing leads, and lead reversals could be erroneously incorporated into DNN algorithms. To overcome this problem, saliency maps were created to make the internal black box process more understandable. Specifically, heat maps identify the image points being analyzed; therefore, the experimenter can tell if the artificial intelligence model erroneously focuses on irrelevant points in an image. But a recent study, published in *Nature Machine Intelligence*, shows that saliency heat maps are not yet applicable [78]. Furthermore, the problem of overfitting can also create incorrect input data. Overfitting occurs because the machine recognizes features as only random properties. This problem mainly occurs when the input data are not generalizable to the entire population and are more specific to a single location where they are collected [79]. In the context of electrocardiography, 12 leads may contain redundant input data, leading to an overfitting problem. Lai et al. addressed the overfitting problem associated with 12-lead ECGs. They investigated how to eliminate the redundancy problem of 12-lead ECGs to improve the classification of abnormalities detected by the deep learning system. They found an optimal subset of leads that eliminate overfitting and allow for the correct interpretation and diagnosis of arrhythmias [80].

Robustness refers to the system’s ability to recognize misleading data. The diagnosis of arrhythmias by cardiologists is linked to gross features in the ECG tracing, such as the lack of P waves and irregular RR intervals for the diagnosis of AF. While the human eye does not perceive small variations in the tracing, deep learning recognizes these perturbations and creates contradictory examples that lead to incorrect rhythm diagnoses [81]. This problem can undermine the security of the deep learning system. 

Systems intended to operate without human supervision must be able to recognize such perturbations and work well, even with contradictory data. The limitations and vulnerabilities of deep learning systems do not cast a shadow on their use in clinical practice but must be recognized to implement their safe use. Deep learning systems and CNNs need a design based on the safety and reliability of the results obtained, so as to not run into ethical problems associated with black boxes.

## 11. Conclusions

The application of artificial intelligence in cardiology is a useful tool to support professionals and improve the quality of care. Artificial intelligence and machine learning will not put healthcare professionals out of business; rather, they will enable healthcare professionals to do their jobs better and leave time for the human–human interactions that make medicine the rewarding profession we all value. One hypothesis could be the use of AI for double-checking diagnoses or as a co-pilot in helping cardiologists in clinical practice. The use of deep learning models for ECG interpretation is a practice that is growing and evolving greatly. The addition of artificial intelligence to a standard ECG—a widely available, inexpensive, painless test—transforms it into a powerful tool for reducing diagnosis time and continuous monitoring with wearable devices and enabling physician support and disease prediction. 

For example, the interpretation of an ECG with the AI could be used to generate a diagnostic hypothesis, such as left ventricular dysfunction or other pathologies not detectable with the standard interpretation of the tracing.

The challenge is to optimize such a tool to overcome limitations related to ethical issues, raw input data, misleading inputs, overfitting, and black boxes. The goal is to make the use of deep learning algorithms reliable and safe without human supervision to enable wide application of such mechanisms that can revolutionize modern medicine in the prevention, diagnosis, and treatment of disease.

## Figures and Tables

**Table 1:** Recent studies related to AI applied to ECGs.

Field of Application	Authors	Disease Detected	AUC	Sensitivity (%)	Specificity (%)
Atrial fibrillation	Attia et al. [21]	Afib during sinus rhythm	0.87	79	79
	Raghunath et al. [22]	New-Onset Afib	0.85	69	81
	Dorr et al. [23]	Afib using smart watch	0.93	94	98
	Guo et al. [24]	Afib using smart watch	-	93	84
	Tison et al. [25]	Afib using smart watch	0.97	98	90
Valvulopathies	Cohen-Shelly et al. [9]	AS	0.85	78	74
	Kwon et al. [26]	AS	0.87	80	79
	Harmon et al. [27]	AS progression	-	78	74
	Kwon et al. [28]	MR	0.84	90	61
Ventricular dysfunction	Attia et al. [29]	HFrEF	0.93	86	86
	Adedinsewo et al. [30]	HFrEF	0.89	74	87
	Vaid et al. [31]	LV/RV dysfunction	0.84	76	76
Cardiomyopathies	Rahman et al. [32]	HCM	0.85	90	90
	Ko et al. [33]	HCM	0.96	87	91
	Tison et al. [34]	HCM	0.91	-	-
		PAH	0.94	80	90
		CA	0.86	-	-
		MVP	0.77	-	-
Myocardial infarction	Acharya et al. [35]	MI	-	95	94
	Liu et al. [36]	MI	-	95	97
	Baloglu et al. [37]	MI	-	99	-
	Lodhi et al. [38]	MI	-	94	86
	Chen et al. [39]	MI	0,99	-	99
Ischemic cardiomyopathy	Gumpfer et al. [40]	Myocardial scar	0.89	70	84
Electrolyte abnormalities	Galloway et al. [41]	Hyperkalemia	0.86	90	58
	Lin et al. [42]	Hyperkalemia	0.96	83	98
		Hypokalemia	0.93	97	93
	Attia et al. [43]	Bloodless K^+^ Determination	-	-	-

**Table 2:** Obstacles and challenges related to AI. Definition of explainability, uncertainty, and robustness.

	Explainability	Uncertainty	Robustness
Obstacle	The inability to monitor the mechanism of black boxes and correct the risk of unreasonable decisions leads to important ethical problems.	Uncertainty error is related to the use of raw data, which increases the amount of noise. Overfitting occurs when input data are not generalizable to the entire population and are more specific than a single location where they were collected.	Misinterpretation of misleading data that are misclassified.
Challenge	Explainable artificial intelligence would make the machine’s decision-making process known, allowing ethical problems to be overcome.	The quantification of uncertainty is crucial to increase confidence in the results obtained.	Robust model of correct recognition of contradictory input for accurate and correct classification.
